# Erie County Society Proceedings and Niagara University

**Published:** 1884-05

**Authors:** 


### Editorial.

ERIE COUNTY SOCIETY PROCEEDINGS AND NIAGARA UNIVERSITY.

We feel that an apology is due to our readers in other States than New York for admitting to our pages in this number so much matter of comparatively local interest. As, however, two of the editors of this journal are connected with the Niagara University, we could not epitomize or select from the proceedings of the Erie County Society without laying the JOURNAL open to the charge of partiality or of allowing personal interests to control its policy.

As the recognized organ of the whole medical profession in Western New York (and not of any clique or college), the columns of the JOURNAL are always open to both sides in any controversy, or on any question of interest to medical men. The JOURNAL, under its present editorial control, has not been slow to condemn a wrong or uphold the right, but on the question before the Erie County Society, for the reasons above referred to, we prefer to publish the official proceedings and to lay the whole matter before our readers without editorial comment.

We are heartily glad, however, to be able to announce that ANY POSSIBLE QUESTION AS TO THE VALIDITY OF THE CHARTER OF THE NIAGARA UNIVERSITY IS FOREVER SET AT REST by a special act which gives the medical department every power it desired, and which, having passed both houses of the legislature, received the Governor's assent on the 3d inst. We take especial pleasure in calling attention to the following among the provisions of the bill, which, it will be noticed, are precisely those contained in the first announcement of the college:

"And the trustees of said college may grant and confer the degree of Doctor of Medicine, but only upon the recommendation of the Board of Medical Professors of said college and of at least three curators of the medical profession fully and legally qualified to practice medicine and surgery, to be appointed by said trustees, and which curators shall not in any manner be connected with said college; but no person shall receive a diploma conferring such degree unless he be of good moral character, and of the age of twenty-one years, and unless he shall have received a good English education, and shall have pursued the study of medicine and the sciences connected therewith at least three years after the age of sixteen years, and received instruction from some physician and surgeon legally authorized to practice his profession, until he is qualified to enter a medical college, and shall also, after that age, have attended at least three complete courses of lectures in the medical department of some legally incorporated university or medical school, the last of which courses shall have been so attended in the medical department of said college."

We have not space in this number for the favorable comment which this bill deserves. The Niagara University has not only put an end to the injurious reflections cast upon the legal standing of its medical department, but by its course in adopting and pledging itself to sustain a curriculum in advance of that of any college in the State, it should and will receive the cordial support of the profession at large. We hope that the Buffalo Medical College will have the courage to adopt the same high requirements from the candidates for its diplomas. The medical colleges of Western New York will then be a credit to the State and country.

